# 25+ Years of TRPV4: From Discovery to Translational Horizons

**DOI:** 10.21203/rs.3.rs-10022857/v1

**Published:** 2026-06-16

**Authors:** Wolfgang Liedtke

**Affiliations:** Duke University

**Keywords:** TRPV4, TRPV4 channelopathy, TRPV4 translational research, TRPV4 ion channel

## Abstract

Transient receptor potential vanilloid member 4 (TRPV4) is a calcium-permeable, nonselective cation channel first described in 2000. Polymodally-activated, also by osmotic, mechanical, thermal, actinic, and chemical stimuli, TRPV4 has emerged as a versatile molecular integrator across a remarkable diversity of cell lineages and vertebrate organ systems. Re orthology to invertebrate TRPV channels, TRPV4 rescued osmotic and mechanical sensing in ASH nociceptor neurons of *C. elegans* mutant for OSM-9 TRPV channels. This chapter surveys the arc of TRPV4 research from foundational cloning to translational medicine target. For that, TRPV4 expression, channel function, and (patho-)physiological roles as deduced from preclinical models and human disease are discussed. Topics span neurobiology and neuroprotection, pain-itch, inflammation, vascular and cardiac biology, pulmonary and airway function, skin, gastrointestinal tract, renal-urinary and uterine biology, skeletal and connective tissue, metabolism, stem cell biology, tissue engineering, cancer, basic ion channel biology, and hereditary TRPV4 channelopathies affecting skeletal development and peripheral motor innervation. Therapeutic strategies—from small-molecule modulators to lipid nanoparticle-delivered gene silencing—are discussed in the context of examples of endothelial translational paradigms, furthermore TRPV4's impact on sensory function including pain and itch. A brief personal reflection on the discovery of TRPV4 concludes this Systematic Review.

## 1. Introduction and Roots

In October 2000, two research groups independently reported the cloning of a novel member of the transient receptor potential (TRP) superfamily of cation channels. At my own end, we described VR-OAC (vanilloid receptor-related osmotically activated channel) in *Cell*, identifying it as a vertebrate osmoreceptor activated by hypo-osmotic stimuli and expressed in osmoregulatory brain nuclei, peripheral osmo-sensing tissues, and in trigeminal ganglion sensory neurons small-to-medium size, also in Merkel cells surrounding vibrissae (1). The latter feature, together with tuning of the sensitivity of the channel by temperature led us to reason about VR-OAC (TRPV4) being involved in mechanotransduction of somatosensory signaling, including touch and pain. Re thermal tuning of the channel's sensitivity, we noticed a maximum sensitivity of the mammalian channels that we had cloned at 37°C, starkly contrasting with the avian isoform's optimum sensitivity at 40°C, the former observation reminiscent of psychophysical sensory thresholds for touch and vibration in humans, and vibrissal detection of flow in seals (1). Strotmann-et-al. reported OTRPC4, a nonselective cation channel conferring osmosensitivity, in Nature Cell Biology (2). These papers have now been referenced more than 2800 times. These foundational reports established what became known as TRPV4 - the third mammalian vanilloid subfamily member of the TRP channel superfamily, oddly named “V4”. TRPV4 was the first vertebrate TRPV channel to originate from outside the Julius-Lab at UCSF which had co-founded the TRPV family by reporting TRPV1 (then named VR1; and later also TRPV2), with foundational co-contribution by the Bargmann-Lab, also at UCSF, who had reported *osm-9* mutants in *C. elegans* (126).

TRPV4 is a polymodally activated, calcium-permeable cation channel. Its activation repertoire is broad: hypo-osmotic cell swelling, mechanical stress, moderate warmth (> 27°C), ultraviolet B irradiation, and diverse chemical signals including endogenous lipid mediators such as epoxyeicosatrienoic acids, lysophosphatidylcholines, and arachidonic acid metabolites, as well as synthetic agonists such as GSK1016790A and 4α-phorbol didecanoate (1–15). TRPV4 can also be sensitized or directly gated by lipid second messengers generated by PLA2 or PLC signaling, acting downstream of multiple classes of G-protein-coupled receptors, including PAR-2, angiotensin, bradykinin, purinergic, serotonin, acetylcholine, prokineticin, neurokinin, and prostaglandin E2 receptors (8–15). This polymodal integration positions TRPV4 as a versatile molecular sensor at the interface of physical microenvironment and cellular signaling.

Now in its third decade, TRPV4 research has expanded from basic biophysics to a truly multisystem physiology, pathology and translational medical science. The channel is expressed in a range of cell lineages: neurons, glia, endothelia, epithelia, immune cells, chondrocytes, osteoblasts, osteoclasts, keratinocytes, adipocytes, striated muscle including cardiac myocytes, smooth muscle cells, and stem cells, among others (3, 16–45). Expression is potently regulated by the metabolic and endocrine state, mechanical microenvironment, inflammation, and other forms of cellular stress. For example, more than 400 papers on TRPV4 in endothelia alone have been published since the first report of endothelial TRPV4 expression in 2002 (3, 14, 46–52). It is this breadth—and the increasingly clear disease relevance—that makes TRPV4 a compelling subject as TRP channel research enters its fourth decade.

## 2. Expression

TRPV4 expression spans virtually all major organ systems and cellular lineages. In the nervous system, TRPV4 is found in primary sensory neurons of the dorsal root ganglia (DRG) and trigeminal ganglia, hippocampal, cortical, basal ganglia and brainstem neurons, hypothalamic osmosensory nuclei, astrocytes, microglia, retinal ganglion cells, and satellite glial cells (1, 4, 10, 25, 30, 34, 53). In the vasculature, robust expression is documented in endothelial cells of arteries, arterioles, capillaries, and veins—including the specialized endothelia of the blood-brain barrier (BBB), blood-spinal cord barrier (BSCB) and blood-retina barrier (BRB)—and in vascular smooth muscle cells (14, 18, 46–52, 54–57). Epithelial expression includes keratinocytes of the skin, renal tubular epithelia, bladder urothelium, alveolar and airway epithelia, cholangiocytes, pancreatic ductal cells, and the lining epithelia of the oral and nasal cavity including sinuses, pharynx, also esophagus, colon and vagina (7, 12, 39–43, 49). In the musculoskeletal system, TRPV4 is expressed in articular chondrocytes, osteoblasts, osteoclasts, skeletal muscle, and tendon fibroblasts (5, 31, 32, 58–63), furthermore in cardiac myocytes and specialized excitatory cells of the cardiac pacemaker and conduction system. Immune cells expressing TRPV4 include macrophages, neutrophils, T cells, mast cells, dendritic cells and NK cells (21, 37, 50, 64). Adipocytes of both white and brown adipose tissue express TRPV4, linking the channel to metabolic regulation (44). This remarkable breadth of expression underpins TRPV4’s diverse physiological roles and its involvement in numerous disease processes (3). Of note, under different conditions - disease states including inflammation, injury, recovery, endocrine exposures and changes, all forms of stress, including mechanical, thermal, oxidative, and more - the channel can be upregulated in lineages where under physiologic circumstances and in normal development expression is feeble.

## 3. Channel Function

**Figure 1:**
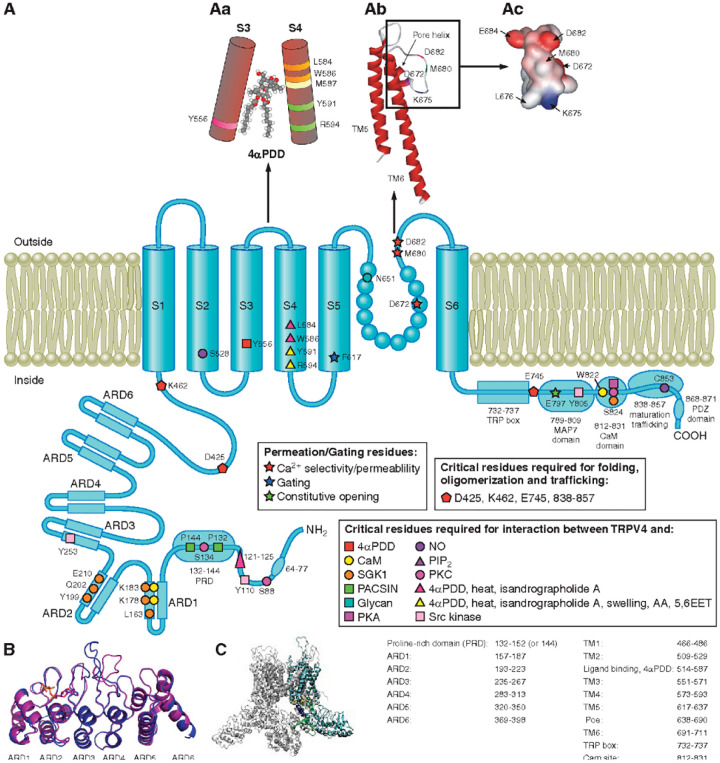
TRPV4 channel protein schematic with relevant AA residues and domains A) Panel shows a schematic of the ion channel protein within the membrane, with highlighted key residues and protein subdomains of the channel. Aa subpanel depicts 4-alpha-PDD binding site between transmembrane domain S3 and S4. Ab subpanel shows the “heart of the channel”, selectivity filter and pore between S5 and S6, structural schematics, helix model. Ac subpanel depict the subdomain of the pore helix K675-E684, structural model with charge landscape, positive charge in blue, negative charge in red. B) Panel shows the helical structural rendering of N-terminal ankyrin repeats 1–6. C) panel shows the channel tetramer, side view, helical model, 2 channel subunits of one tetramer depicted. Tabulated: residues and domains, position within the channel protein (human channel). From (3).

TRPV4 is a nonselective cation channel with a preference for calcium over monovalent cations (PCa^2+^/PNa^+^ ≈ 6–10). The channel assembles as a homotetramer, with each subunit contributing six transmembrane domains, a pore loop between S5 and S6, and intracellular N- and C-termini containing functionally important ankyrin repeat domains, a calmodulin-binding site, and regulatory phosphorylation sites (3, 13), see Fig. 1. Cryo-electron microscopy structures have revealed conformational states associated with channel gating and have informed understanding of how diverse stimuli converge on pore opening.

The polymodal activation of TRPV4 is a defining feature. Osmotic activation proceeds through phospholipase A2-dependent generation of arachidonic acid and its cytochrome P450 epoxygenase metabolites, particularly 5,6-epoxyeicosatrienoic acid (14, 15). Mechanical activation can occur rapidly via direct membrane-force sensing through basal membrane distension at the cell matrix interface and also via indirect cytoskeletal coupling, an experimentally verified mechanism, yet benefitting from additional experimental inquiry, and importantly depending on cellular context (5, 20, 23): in other words, TRPV4 can function as a direct mechanotransductory channel in one type of cell, not in another one, and importantly to some submodalities of mechanical cues, not to others (5, 20). Thermal sensitivity in the warm range (> 27°C) has been characterized in heterologous expression systems and native cells (6). Chemical activation by endogenous glycerophospholipids, including lysophosphatidylcholine, has been demonstrated with evidence for a C-terminal binding site (11, 12). Across virtually all settings, the decisive downstream signal is calcium influx through the channel pore, engaging cell-type-specific signaling cascades including calmodulin, protein kinase C, calcineurin, and cytoskeletal remodeling pathways (3, 13, 29). Beta-arrestin-1 mediates ubiquitination and functional down-regulation, providing a critical negative regulatory mechanism (9). The IP3 receptor binds to and sensitizes TRPV4 to osmotic stimuli via the calmodulin-binding site (13). Interrogation of TRPV4 and PIEZO1 in concerted interaction with PIEZO2 in chondrocytes has revealed coexisting mechanotransduction pathways that typically synergize (5). Of note, urothelia in the urinary bladder also share this mechanosensitive ion channel expression pattern and synchronized function between TRPV4 and PIEZO1/2 channels (139).

TRPV4 channel function is preserved in vertebrates. TRPV4 was found orthologous to invertebrate OSM-9 TRPV function in *C. elegans* (4, 24). In worms’ head nociceptor ASH neurons, the worm’s single neuron equivalent to vertebrate trigeminal ganglia, mammalian TRPV4 could rescue sensory deficits for sensing of aversive osmotic and mechanical, not chemical cues, in *osm-9* mutant worms. In noteworthy follow-up, Amanda Lindy and colleagues from my former laboratory investigated how OSM-9 ion selectivity governs worms’ avoidance behavior by examining calcium transients in ASH neurons (129). By systematically mutagenizing the OSM-9 selectivity filter (M601–F609) we were able to decouple calcium permeation from sodium conductance and avoidance behavior. The Y604F mutant of OSM-9 abolished measurable calcium transients yet preserved robust osmotic avoidance—mediated instead by sodium influx and neuronal depolarization—while the Y604G mutant eliminated both calcium flux and behavior. Strikingly, the Y604F worms failed to desensitize upon repeated noxious stimulation, revealing the important general concept that calcium entry through the pore of a TRPV channel drives survival-relevant behavioral plasticity, namely habituation, rather than actuating acute and monotonic withdrawal when faced with an aversive stimulus. The Lindy-et-al. study together with the earlier demonstration of TRPV4-OSM-9 orthology (4, 24) guide us re a fundamental question of TRP ion channel function: sodium influx is sufficient for an immediate cellular and subsequent organismal response such as avoidance, whereas calcium influx subserves longer-term adaptive modulation, both enhancing organismal survival value. These findings support the concept that distinct ionic permeation modes through the channel pore fulfill separable physiologic roles and provide an experimental answer to the question of how the TRP family has evolved as calcium-permeable channels.

## 4. Physiological and Pathophysiological Roles

### 4.1 Neurobiology and Neuroprotection

TRPV4 has roots in neurobiology, beginning with its initial identification in osmosensory brain regions (1). In the hypothalamus, TRPV4 contributes to central osmoregulation and vasopressin release, and *Trpv4*^*−/−*^ mice display abnormal osmotic regulation with impaired behavioral responses to osmotic challenge (4, 24, 25). Beyond the hypothalamus, TRPV4 is expressed in hippocampal neurons where it modulates synaptic transmission and neuronal excitability, with its tuning by body temperature (121); its activation enhances glutamate-driven network activity, while its inhibition or genetic deletion attenuates excitotoxic injury (53). In the brainstem, TRPV4 function is critical for astrocyte mechanosensitivity, where it acts as a sensor of membrane stretch. Upon activation, TRPV4 channels functionally interact with connexin 43 (Cx43) hemichannels, triggering the release of ATP that propagates calcium signals and provides excitatory drive to brainstem circuits controlling heart rate (157).

Astrocytic TRPV4 has emerged as a key regulator of brain volume homeostasis and neurovascular coupling (30, 53). At perivascular endfeet, TRPV4-mediated calcium signaling triggers the release of vasoactive messengers that match local blood flow to neuronal activity, a process critically dependent on TRPV4’s concerted interaction with aquaporin-4 (30). Microglial TRPV4 participates in inflammatory activation, phagocytosis, and regulation of pain at the spinal level (50, 53). In the retina, TRPV4 modulates calcium flux, spiking rate, and apoptosis of retinal ganglion cells, with implications for glaucoma and retinal degeneration (34); it also contributes to the mechanosensitivity of Müller glial cells (34). TRPV4 additionally contributes to endoplasmic reticulum stress and inflammation with implications for Parkinson’s disease (40). Emergent roles for TRPV4 in oligodendrocyte differentiation and myelination further underscore its broad relevance to CNS development and white matter integrity (158).

**Figure 2:**
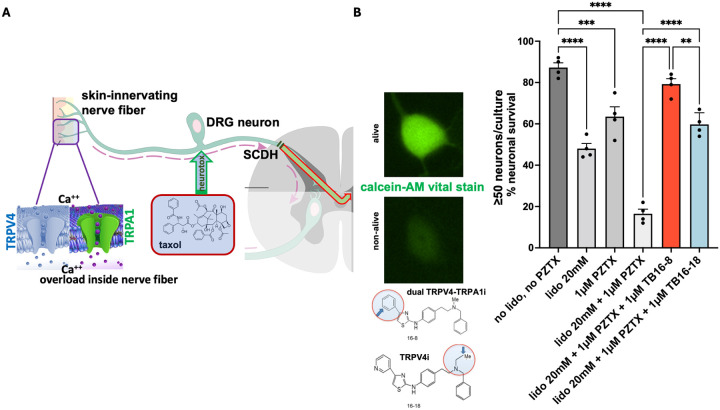
TRPV4 in sensory neuronal function - example chemotherapy-induced painful neuropathy (CIPN), based on data from (53,130). A) Panel depicts pathogenesis of CIPN co-dependent on TRPV4 and TRPA1 overexpression in DRG sensory neurons, as they are injured by exposure to chemotherapeutic agent, paclitaxel. B) Shows results of primary DRG neuron cultures from mouse examining their viability, and injuring them with paclitaxel exposure, and to enhance paclitaxel toxicity, with lidocaine. Bar diagram on the right shows survival in response to toxicity, note potent impact of lido + paclitaxel, appreciable rescue by the selective TRPV4-inhibitor, compound 16–18 (a derivative of tool compound GSK205), and powerful rescue by potent dual TRPV4-TRPA1-inhibitor, compound 16–8. Survival with protection by compound 16–8 is statistically not significantly different from sham exposure, indicating complete rescue.

Neuroprotective functions of TRPV4 modulation were also demonstrated by compound-mediated inhibition of TRPV4 which greatly reduced neuronal demise of cultured DRG neurons exposed to lidocaine and paclitaxel (Fig. 2), with enhanced protection afforded by dual TRPV4/TRPA1 inhibitor compound 16 − 8—rescuing survival from 16.5% to approximately 79% (130, 121). These findings carry direct implications for chemotherapy-induced and local anesthetic neurotoxicity.

In another translationally-relevant finding involving TRPV4's role in neuroprotection, Luo-et-al. demonstrated that TRPV4 loss-of-function or pharmacological inhibition attenuates neurotoxicity of the chemical nerve warfare agent, soman (131). *Trpv4*^−/−^ mice showed reduced seizure burden with delayed onset and markedly decreased lethality. Mechanistically, TRPV4-mediated calcium influx drove phosphorylation of the glutamate receptor subunit NR2B and activation of the NLRP3 inflammasome in hippocampal neurons; blocking TRPV4 was neuroprotective against soman-induced cell death. These observations implicate TRPV4 as a therapeutic target in excitotoxic and inflammatory neuronal injury.

### 4.2 Pain: Mechanical, Inflammatory, Trigeminal, and Visceral

TRPV4’s role in pain signaling was appreciated from its earliest characterization as a candidate nociceptive transducer, giving a robust in-situ hybridization signal in small-to-medium size trigeminal ganglion neurons (1). In inflammatory pain, TRPV4 sensitization downstream of protease-activated receptor 2 (PAR-2) contributes to mechanical hyperalgesia and neurogenic inflammation (8, 38). Cathepsin-S causes inflammatory pain via biased agonism of PAR-2 and TRPV4 (38). Trigeminal TRPV4 has been implicated in orofacial pain, temporomandibular joint pain, and dural afferent activation relevant to headache (28, 36, 57, 65). TRPV4 was shown to function as a cellular formalin receptor necessary for trigeminal irritant pain (65). Visceral pain pathways engage TRPV4 in sensory neurons innervating colon, pancreas, and bladder, where the channel transduces distension and inflammatory signals into nociceptive afferent activity (16, 27, 42, 66). TRPV4 was also found to function pro-pain in vulvodynia, a disabling form of female sexual and pelvic pain, where TRPV4 enhanced a pro-inflammatory, pro-algesic phenotype of vulvar fibroblasts of vulvodynia women (132). In the skin, keratinocyte TRPV4 mediates UVB-induced sunburn pain through endothelin-1 signaling (7, 12). These diverse pain paradigms underscore TRPV4 as a “pain-TRP” with broad therapeutic potential.

### 4.3 Inflammation

TRPV4 participates in both innate and adaptive immune responses across multiple cell types. In macrophages, TRPV4 regulates LPS-stimulated phagocytosis, matrix stiffness-induced polarization, and inflammatory signaling relevant to the tissue microenvironment (50, 60, 64). Neutrophil TRPV4 contributes to acute lung injury via reactive oxygen species generation and chemotactic responses (37). The mechanosensory function of TRPV4 has been proposed as a missing link between mechanosensation and immunity, as tissue stiffening during inflammation can facilitate TRPV4 activation in immune cells, amplifying the inflammatory response in a feed-forward loop (21). In the vasculature, endothelial TRPV4-mediated calcium influx is a key event in inflammatory endothelial activation, contributing to increased permeability, leukocyte adhesion molecule expression, and transendothelial immune cell migration (68, 69). TRPV4 also plays a significant role in tonicity-induced neurogenic inflammation (67).

### 4.4 Vascular Biology and Heart

Endothelial TRPV4 is a master regulator of vascular function. The channel mediates mechanotransduction in response to shear stress and intravascular pressure, metabolic interfacing between endothelia and vascular smooth muscle, capillary barrier formation, angiogenesis, vascular maintenance and aging, and blood pressure regulation (14, 18, 46–52, 55–57, 70, 71). Elementary calcium sparklets through endothelial TRPV4 channels were shown to regulate vascular function (56). In arteriolar endothelia, TRPV4 is physiologically phosphorylated by protein kinase-C orchestrated by the AKAP150 scaffolding protein. TRPV4-mediated calcium influx activates adjacent IK/SK calcium-activated potassium channels, which hyperpolarize the endothelial microdomain; this hyperpolarization is transduced via myo-endothelial gap junctions to vascular smooth muscle cells, where it inhibits voltage-gated calcium channels and promotes vasorelaxation (54, 56).

In obesity-related hypertension, this physiological mechanism is impaired by excessive peroxynitrite (O = NOO^−^) generated by endothelial NADPH oxidase 1 (NOX1), which is overexpressed in obesity. Peroxynitrite annihilates the AKAP150-PKC coupling, TRPV4 channels are no longer phosphorylated and become hypofunctional, allowing lack of potassium efflux via IK/SK channels, which depolarizes vascular smooth muscle resulting in hypertension. These mechanistic findings were ratified in explanted human arterioles from splenius and temporalis muscles of obese patients (54). This finding represents a paradigmatic example of TRPV4 endothelial hypofunction contributing to a clinically relevant facet of obesity. TRPV4 also regulates collateral vessel growth during arterial regeneration and endothelial cell reorientation in response to cyclic strain via integrin-to-integrin signaling (70, 71).

In cardiopulmonary fibroblast lineage, TRPV4 has been implicated in myofibroblast differentiation and pulmonary fibrosis (29, 51).

**Figure 3:**
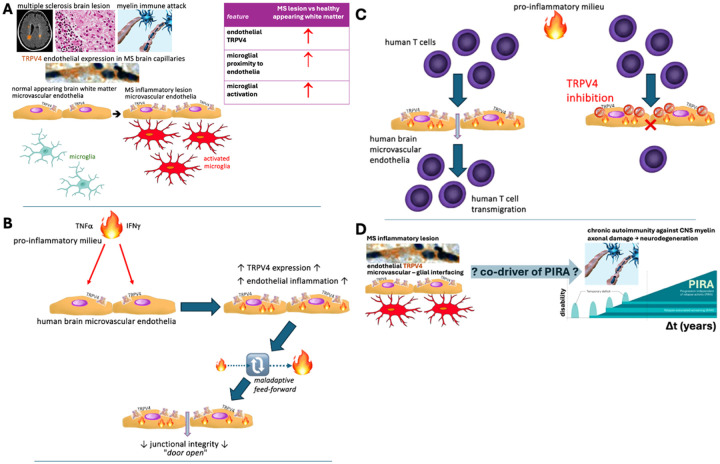
TRPV4 expression and function in vascular endothelia - example Multiple Sclerosis TRPV4 in blood brain barrier endothelia in multiple sclerosis enhancement of endothelial inflammation and cross-talk to microglia possible role in MS PIRA – progress independent of relapse activity. From (121). A) TRPV4 expression in human endothelia in MS brains, their role in activating nearby microglia. B) Peri-endothelial inflammation, via IFNg and TNFa, lead to “endotheliitis” encompassing a leaky barrier. C) TRPV4 pharmacological inhibition blocks T-cell transmigration through the endothelial barrier as it faces an inflammatory microenvironment. D) TRPV4 in human brain endothelia - a role in PIRA ?

For exemplary endothelial expression of TRPV4 in Multiple Sclerosis, see Fig. 3.

### 4.5 Lung and Airway Biology

TRPV4 plays critical roles in both upper and lower airway biology and in the alveolar barrier. In the lungs, TRPV4 activation in endothelia and alveolar epithelia increases permeability and can precipitate lethal pulmonary edema; conversely, TRPV4 blockade prevents ventilator-induced lung edema and resolves pulmonary edema induced by heart failure (15, 72–78). Potent systemic activation of TRPV4 causes endothelial failure and circulatory collapse, underscoring the channel’s impact on barrier regulation (73). A negative-feedback loop via cGMP-dependent regulation has been identified as a physiological brake on hydrostatic lung edema (72). Lung microvascular endothelial TRPV4 is activated by mechanical stretch during high-tidal-volume ventilation and by chemical mediators including arachidonic acid metabolites, leading to calcium-dependent endothelial barrier disruption and increased vascular permeability (75, 76). TRPV4 inhibition attenuates pulmonary edema in rodent models of ventilator-induced and chemical-induced lung injury (74, 77). Furthermore, TRPV4 signaling in pulmonary endothelial cells contributes to the development of pulmonary hypertension: TRPV4-mediated calcium influx promotes endothelial dysfunction and the release of proliferative and vasoconstrictive mediators that drive pulmonary vascular remodeling (159). Beyond the lung endothelium, TRPV4 has documented roles in the coronary microvasculature, where it mediates flow-dependent vasodilation (56), and in the mesenteric circulation, where it contributes to myogenic tone and pressure-induced constriction (160), highlighting its broad significance across multiple vascular beds.

TRPV4 is required for hypoxic pulmonary vasoconstriction, linking the channel to ventilation-perfusion matching (79). TRPV4 can protect the lung from bacterial pneumonia via MAPK molecular pathway switching, demonstrating context-dependent protective versus pathogenic roles (33). Inflammatory pulmonary diseases engage TRPV4 in neutrophils, alveolar macrophages, and vascular endothelia (37, 43, 80). TRPV4 has also been implicated in cystic fibrosis airway pathology, where (i) swelling-activated calcium entry via the channel is defective (49), (ii) TRPV4 enhances inflammation also by neutrophil recruitment and hyperproduction of pro-inflammatory lipids, (iii) TRPV4 activation in airway epithelia can enhance mucus clearance, (iv) defective CFTR impacts TRPV4 as gatekeeper of endothelial barrier integrity. In human bronchial epithelial cells, TRPV4 mediates calcium influx in response to diesel exhaust particle exposure, adding an air pollution-driven airway injury mechanism to its functional repertoire (124).

### 4.6 TRPV4 in Tuberculosis and Globally Relevant Infectious Diseases

An underappreciated dimension of TRPV4 biology is its role at the intersection of ion channel physiology and host-pathogen interaction, with particular significance for tuberculosis.

TRPV4 plays a biphasic role in TB. Naik-et-al. showed that virulent Mtb suppresses TRPV4 in macrophages within 24 hours, redistributing it from the plasma membrane (133). In TRPV4-deficient macrophages, IFN-γ fails to induce phagosome acidification, nitric oxide production, and Mtb delivery to lysosomes, leading to necrotic death — defining TRPV4-mediated calcium flux as critical for phagolysosomal maturation in TB (133).

In Trpv4−/− mice, these defects cause higher pulmonary Mtb burden at day 29. However, by day 150, the phenotype reverses: Trpv4−/− mice show lower Mtb burden, reduced inflammation, fewer foamy macrophages, and less neutrophil elastase accumulation. Elevated IL-10 and diminished lipid bodies may further restrict bacterial growth. In human granulomas, TRPV4-positive cells localize peripherally, with reduced expression in necrotic centers (133).

Sundaramurthy-et-al. identified a complementary mechanism: Mtb sulfolipid-1 activates TRPV4 to drive mTORC1–TFEB-dependent lysosomal biogenesis and exocytosis (134). Thus, TRPV4 is acutely host-protective yet chronically detrimental by driving immunopathology and Mtb-permissive lipid accumulation in macrophages. This temporal dissociation suggests stage-specific therapeutic targeting — transient potentiation early to enhance clearance, antagonism later and chronically to limit tissue destruction.

Beyond TB, TRPV4’s roles in macrophage polarization, neutrophil function, and epithelial barrier integrity are relevant to broader infectious diseases. In pulmonary infections, TRPV4-mediated regulation of alveolar permeability and macrophage-driven cytokine production places it at a key node in innate defense. TRPV4-dependent stiffness-sensing macrophage polarization — where stiffer substrates drive inflammatory activation — adds a mechanobiological dimension to its immunological functions (33, 50).

TRPV4 is also exploited by dengue, hepatitis C, and Zika viruses. Doñate-Macían-et-al. demonstrated that TRPV4 interacts with the RNA helicase DDX3X (135). Viral envelope proteins trigger TRPV4-mediated calcium influx, releasing DDX3X for nuclear translocation via calmodulin and CaMKII. There, DDX3X facilitates nuclear export of unspliced viral RNA and cytoplasmic translation. TRPV4 inhibition reduces infectivity of all three viruses, positioning TRPV4 antagonism as a potential broad-spectrum adjunct antiviral strategy.

### 4.7 Skin Biology

In the skin, TRPV4 is expressed prominently in keratinocytes, also in dermal fibroblasts, melanocytes, Merkel cells and cutaneous sensory nerve endings (7, 12, 26, 35, 39). Keratinocyte TRPV4 is essential for barrier function, immune signaling, and sensori-neural cross-talk. UVB-induced activation of epidermal TRPV4 contributes to sunburn pain involving TRPV4-dependent endothelin-1 secretion from keratinocytes (7). Integumental macrophage TRPV4 mediates stiffness-induced foreign body response and giant cell formation via Rac1 mechanotransduction (17, 23). TRPV4 is also implicated in wound healing, keratinocyte migration, dermal myofibroblast differentiation relevant to fibrosis and scleroderma, and imiquimod-induced itch, a valid preclinical psoriasis model (26, 29, 35, 67), furthermore in rosacea pathogenesis likely via TRPV4 expression in cutaneous mast cells (26, 125), also keratinocytes (136), upregulated via rosacea-driving LL37 peptide fragment of excessively expressed cutaneous cathelicidin (26). Suppression of TRPV4 function inhibits inflammatory fibrosis in alkali-burned corneas, extending skin biology insights to ocular surfaces (41). These findings collectively establish TRPV4 as a clinically relevant, yet translationally under-leveraged multifunctional integrator in cutaneous physiology and pathology where topical application of TRPV4-targeting therapeutics is readily feasible.

### 4.8 Gastrointestinal Tract

TRPV4 is expressed throughout the gastrointestinal tract in sensory neurons, both enteric nervous system as well as DRG neurons, lining epithelia, and immune cells. In the colon, TRPV4 mediates visceral hypersensitivity and contributes to inflammatory bowel disease pathophysiology (16, 27). Pancreatic TRPV4 participates in pancreatitis-associated pain via neurogenic mechanisms and in exocrine pancreatic ductal ATP release and hydrolysis (42, 66). In the liver, TRPV4 activation of endothelial nitric oxide synthase resists nonalcoholic fatty liver disease by blocking CYP2E1-mediated redox toxicity (52). In the oral cavity and esophagus, TRPV4 is expressed in epithelial linings with relevance for barrier integrity and epithelial–sensory neuron cross-talk, as in skin (12). GI TRPV4 biology encompasses barrier function, secretion, pain, and inflammation.

### 4.9 Renal and Urinary Biology

TRPV4 is appreciably expressed in the distal nephron, where it functions as a polymodal sensor of mechanical and osmotic cues (137). In the collecting duct, it localizes to the apical membrane of aquaporin-2-positive principal cells and intercalated cells, triggering calcium influx in response to flow or hypotonicity that regulates cell volume and potassium secretion via BK channels (138). In glomerular podocytes, TRPV4 helps maintain the filtration barrier and actin cytoskeleton integrity (45). At the juxtaglomerular apparatus, the channel mediates pressure-induced calcium entry that can inhibit renin secretion (137). Critically, TRPV4 forms a heteromeric complex with TRPP2 (Polycystin-2) in the primary cilium to sense fluid flow (137). Dysregulation of this complex, or loss of TRPV4 mechanosensitivity, elevates cAMP and drives cystogenesis in polycystic kidney disease, while pharmacological TRPV4 activation slows cyst growth. In the bladder, urothelial TRPV4 transduces stretch into afferent signaling for normal voiding; its overactivation contributes to overactive bladder and interstitial cystitis (139).

### 4.10 TRPV4 in Uterine Function and Preterm Birth

In the myometrium, TRPV4 expression increases with gestation, enhancing uterine excitability (140). The channel acts as a critical osmo-mechanosensor, and its activation promotes myometrial contractility. Crucially, premature activation or overexpression of TRPV4 is implicated in the onset of preterm labor, linking mechanical stretch and inflammation to untimely contractions. Pharmacological blockade of TRPV4 has been shown to quell uterine contractility in validated preclinical models including human-derived, presenting a rationally-based therapeutic strategy (141). Targeting TRPV4 therefore offers a much needed translational avenue to delay premature delivery and manage this significant global health challenge.

### 4.11 Metabolism, Diabetes, and Obesity

TRPV4 is expressed in white and brown adipose tissue, where it modulates adipocyte differentiation, thermogenesis, and level of inflammation (142). In enteroendocrine L-cells, found in the small intestinal epithelial lining, TRPV4 couples with the sodium-calcium exchanger NCX1 to mediate glucose-dependent glucagon-like peptide-1 (GLP-1) release, identifying a novel TRPV4-NCX1 axis in incretin physiology (44). The intersection of TRPV4 with obesity-related vascular dysfunction as explained above (section 4.4) places the channel at the nexus of metabolic syndrome and cardiovascular disease (54). Whether new incretin-based bariatric therapeutics such as semaglutide, tirzepatide, and retatrutide, which can potently lower body mass index (by more than 10–20%), can reverse obesity-related TRPV4 endothelial impairment represents a now testable clinical question (81–85). In pancreatic beta-cells, activation of TRPV4 causes a rapid influx of calcium, which enhances glucose-stimulated insulin secretion, positioning TRPV4 as a modulator of glycemic control (161). Within the hypothalamus, TRPV4 is expressed by the majority of vasopressinergic neurons where it functions as an osmosensor; its activation regulates vasopressin secretion (25). Furthermore, in the renal juxtaglomerular cells, TRPV4 mediates pressure-induced calcium entry that in turn inhibits renin secretion, as mentioned above (section 4.9), thereby forming a critical feedback loop for blood pressure regulation. Finally, TRPV4 expression appears itself hormonally regulated by the progesterone receptor in various epithelial and vascular tissues (140).

### 4.12 TRPV4 in Biological Fluid Production and Pressure Homeostasis: Cerebrospinal Fluid, Aqueous Humor (Eye), and Endolymph (Ear)

TRPV4 regulates production and pressure homeostasis of specialized biological fluids across three compartments by coupling osmotic and mechanical signals to transepithelial transport.

In the brain’s choroid plexus, TRPV4 mediates osmosensory control of cerebrospinal fluid secretion; its disruption perturbs CSF homeostasis, potentially contributing to communicating hydrocephalus and idiopathic intracranial hypertension (162). In the eye, TRPV4 is expressed in the trabecular meshwork and non-pigmented ciliary epithelium, regulating aqueous humor outflow resistance. Dysfunction is linked to elevated intraocular pressure and primary open-angle glaucoma, with TRPV4 acting as a mechanosensor in this pressure-responsive tissue (143, 144). In the inner ear, TRPV4 localizes to the stria vascularis, the epithelium generating potassium-rich endolymph. While less characterized in the inner ear, its osmotic and mechanical sensitivity suggests a role in endolymphatic homeostasis, with implications for endolymphatic hydrops and sensorineural hearing loss (145, 146).

Across these compartments, TRPV4 functions as a mechano-osmotic sentinel at secretory and drainage epithelia, integrating physical signals into fluid homeostatic responses. Compromise of this function—whether by mutation, inflammatory sensitization, or pathological pressure loading—produces disordered fluid dynamics with direct clinical consequences.

### 4.13 Skeletal Biology: Bone, Cartilage, Joints, and Muscle

**Figure 4:**
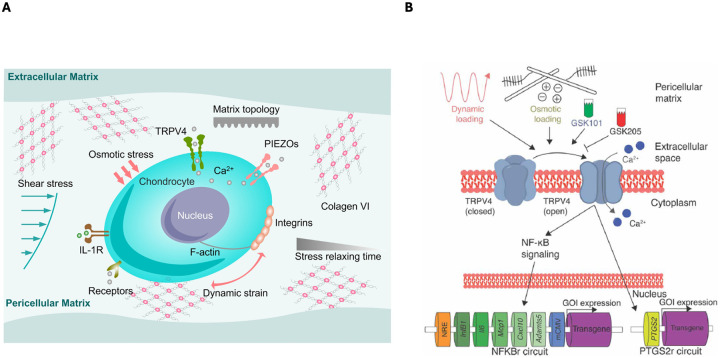
TRPV4 function in chondrocytes – mechanogenetics A) Panel depicts TRPV4 function in articular chondrocytes, with osmo-mechanical activation of the channel supporting pro-anabolic programs of the cell. From (122). B) Mechanogenetics: Mechanical activation of a TRPV4-expressing cells leads to activation of a gene-of-interest via transcriptional drivers that respond to TRPV4-mediated calcium influx and its subsequent cytoplasmatic signaling cascade. Following this principle, a “disease-defeats-itself” approach can be engineerd. From (123).

TRPV4 is a critical mechanosensor in the musculoskeletal system. In articular chondrocytes, TRPV4 mediates mechanotransduction in response to dynamic loading, regulating metabolic responses and matrix homeostasis (5, 32). Matrix viscoelasticity—the time-dependent mechanical properties of the extracellular matrix—additionally tunes TRPV4-dependent mechanobiological responses in chondrocytes (122), Fig. 4. Cartilage-specific knockout of TRPV4 decreases age-related osteoarthritis, demonstrating that tonic TRPV4 activity contributes to degenerative joint disease (59). Synergy between TRPV4, Piezo1, and Piezo2 channels in chondrocyte mechanotransduction has been observed (5, 61), and inflammatory signaling sensitizes Piezo1 mechanotransduction in articular chondrocytes as a pathogenic feed-forward mechanism in osteoarthritis (62). In osteoblasts and osteoclasts, TRPV4-mediated calcium influx regulates terminal differentiation and bone remodeling (31). TRPV4 has been termed the sixth sense of the musculoskeletal system, reflecting its central role in how connective tissues perceive and respond to their mechanical environment (60, 63).

### 4.14 TRPV4 in Skeletal Pathology: Giant Cell Tumors of Bone and Regional Osteonecrosis

Beyond its established roles in chondrocyte mechanotransduction and osteoblast/osteoclast biology, TRPV4 has emerging relevance in discrete skeletal pathologies characterized by focal tissue destruction and dysregulated bone remodeling.

Giant cell tumors of bone (GCTB) are locally aggressive neoplasms dominated by osteoclast-like multinucleated giant cells and their mononuclear stromal precursors, arising most commonly at epiphyseal locations subject to high mechanical loading. TRPV4 expression has been identified in GCTB stromal cells, where its activation promotes osteoclastogenic signaling cascades. Given that TRPV4 gain-of-function mutations are established drivers of skeletal dysplasia and that osteoclast differentiation is sensitive to TRPV4-mediated calcium signaling, the channel represents a plausible therapeutic target in GCTB (163).

Regional osteonecrosis — encompassing femoral head osteonecrosis (avascular necrosis) and Osgood-Schlatter disease, a traction apophysitis of the tibial tubercle prevalent in adolescent athletes — involves focal ischemic or mechanically induced bone and cartilage injury at sites of high compressive and tensile loading. TRPV4's role as a mechanosensory transducer in both osteocytes and chondrocytes, combined with its sensitivity to hypoxia-associated osmotic shifts, positions it as a candidate mediator of the cellular responses to the aberrant mechanical and metabolic microenvironments characteristic of these conditions. Osteocyte TRPV4 activity, operating through microtubule-coupled NOX2/reactive oxygen species signaling (164), may be particularly relevant to the mechanosensory failure that permits progressive bone death in avascular necrosis.

### 4.15 Mechanogenetics: Engineering TRPV4 as a Mechanically Activated Therapeutic Switch

The mechanogenetics concept, developed by Guilak and Liedtke, engineers cells to couple mechanical stimuli to therapeutic gene expression using TRPV4 as the mechanosensory transducer (123), Fig. 4.

In its foundational Science Advances implementation (123), stem cells expressed a TRPV4-responsive, calcium-sensitive construct driving anti-inflammatory IL-1 receptor antagonist production under NFAT control. Physiological mechanical loading—such as compressive forces on articular cartilage—activated TRPV4-mediated calcium influx, triggering on-demand therapeutic payload delivery precisely at the site and moment of loading (123). This design exploits TRPV4’s direct mechanotransduction at cell-matrix contacts and the intrinsic spatial localization of its activation.

This platform extends the TRPV4 mechanoreceptor framework established by Poole/Lewin pillar array electrophysiology: if TRPV4 is gated directly by matrix-transmitted forces, it can serve as the input transducer for synthetic mechanobiological circuits. Future applications could deploy mechanogenetic constructs for on-demand delivery of analgesics, anabolic growth factors, or disease-modifying biologics in osteoarthritis, intervertebral disc disease, and musculoskeletal injury—conditions where therapeutic need peaks precisely during mechanical loading (5, 123).

### 4.16 Stem Cell Biology and Tissue Engineering

TRPV4 plays a regulatory role in stem cell fate determination, with most insights generated from chondrogenic lineage programming. The channel was identified by functional gene screening as a regulator of chondrogenic differentiation (58), where ECM-interactions control stem cell fate via TRPV4-dependent mechanotransduction (63). In tissue engineering, TRPV4-mediated calcium signaling is leveraged for cartilage tissue construction and astrocyte bioengineering (147). The translocation of TRPV4-PI3Kγ complexes to the plasma membrane drives myofibroblast transdifferentiation, a process relevant to fibrotic tissue remodeling and engineering applications (29). These findings establish TRPV4 as a mechanosensitive node in regenerative medicine and tissue engineering.

### 4.17 Cell Growth and Cancer

TRPV4 has emerged as a candidate co-oncogene and regulator of tumor biology (148). TRPV4 activity drives cell migration and invasion in multiple cancer types, also proliferation. TRPV4-mediated calcium influx contributes to pathological angiogenesis and vascular normalization within the tumor microenvironment (52). TRPV4 activation influences macrophage polarization within the tumor microenvironment and regulates oxidized LDL-induced macrophage foam cell formation relevant to the inflammatory milieu of the tumor stroma (64). The intersection of TRPV4 mechanosensing with the altered mechanical properties of tumor tissue represents an active frontier of cancer biology (148). For many tumor types, though, there appears the translational dilemma that inhibition of TRPV4 could have beneficial impact on tumor cell migration and proliferation, whereas for inhibition of tumor angiogenesis channel activation is desirable (45).

## 5. Relevance for Human Diseases as Rooted in Human Genetics: TRPV4 Channelopathies - Skeletal and Neural

Disease-causing gain-of-function mutations in TRPV4 were discovered more than fifteen years ago and define a growing family of hereditary disorders with mostly nonoverlapping skeletal and neural phenotypes (45, 58, 86–99). The skeletal TRPV4 channelopathies—including autosomal-dominant brachyolmia, spondylometaphyseal dysplasia (Kozlowski type), metatropic dysplasia, and inherited arthropathy of hands and feet—are caused by mutations that evoke excessive calcium influx into developing chondrocytes, driving pathological hypertrophic mal-differentiation (58, 86, 92, 95, 96). Follistatin in chondrocytes has been identified as a mechanistic link between TRPV4 channelopathies and skeletal malformations because the gain-of-function TRPV4 channels drive increased persistent expression of follistatin in chondrogenic precursors, which in turn secrete excessive follistatin, which then potently neutralizes BMP activity in the developing skeleton, at least partially explanatory for the phenotype (86).

The neural TRPV4 channelopathies manifest as Charcot-Marie-Tooth disease type 2C (CMT2C), distal hereditary motor neuropathy, and distal spinal muscular atrophy (dSMA), with progressive motoneuron demise as the hallmark clinical feature (87–94, 97). TRPV4 has been identified as a trigger of pathological RhoA activation in neurological disease (99).

A transformative recent discovery has overturned the prevailing assumption that motoneuron death in TRPV4 neurochannelopathies is exclusively cell-autonomous and rooted in motoneurons. Charlotte Sumner's group, who co-discovered TRPV4 neuro-channelopathy mutations, using humanized knock-in mouse models carrying disease-causing TRPV4 mutations (R269C and R232C), demonstrated that genetic deletion of the mutant TRPV4 allele specifically from endothelial cells—but not from neurons, microglia, astrocytes, oligodendrocytes, or muscle—rescued the progressive motoneuron degeneration, early weakness, and lethality phenotypes in dramatic fashion (100). Gain-of-function mutant TRPV4 in vascular endothelia of the developing spinal cord and brain stem causes excessive Ca^2^^+^ influx that damages endothelial junctional integrity, compromises the blood-spinal cord barrier (BSCB), and allows leakage of neurotoxic systemic mediators, e.g fibrinogen, that program irreversible motoneuron degeneration. Systemic administration of a TRPV4-specific antagonist reversed BSCB permeability defects and strikingly restored ambulation in mutant mice. This paradigm-shifting work provides evidence that motoneuron death in these disorders is driven by vascular—not neuronal—pathology, opening transformative therapeutic strategies (100).

## 6. Relevance for Human Diseases - Exemplary Expression in Endothelia and Sensory Neurons

### 6.1 Endothelial TRPV4 in Multiple Sclerosis

In addition to endothelial TRPV4 driving obesity hypertension (section 4.4), conferring an anti-angiogenesis phenotype in tumor vasculature (4.17), and assuming pivotal roles in permeability of vascular barriers in the BBB, BSCB and BRB (blood retinal barrier), a specific endothelial role for TRPV4 in multiple sclerosis (MS) was recently elaborated, Fig. 3. MS is a relapsing-remitting autoimmune disease of the central nervous system in which leakiness of the blood-brain barrier (BBB) critically contributes to relapse frequency and severity (101–107). TRPV4 is expressed in brain microvascular endothelia, and in active MS lesions, lesional microvascular endothelia show increased TRPV4 expression with accentuated proximity of activated microglia (69). In a human primary culture model of the MS BBB, inflammatory activation by microglia upregulates endothelial TRPV4 with TNFα as a key mediator. In the TNFα and IFNγ inflammatory milieu, microvascular endothelial cells switch to a pro-inflammatory program, relying on TRPV4. Exposing cultured endothelial cells to TNFα and IFNγ increased T cell transmigration, which was significantly attenuated by selectively inhibiting TRPV4 (69). An attractive pathophysiologic concept emerges: the development of progression independent of relapse activity (PIRA)—a current “hot topic” in MS translational research (105, 108–113)—may be related to endothelial inflammation as a co- driver of chronic glial activation in the MS brain, with Ca^2+^ influx via TRPV4 in endothelia functioning centerfold. Clinically feasible biomarkers of early PIRA, including retinal synaptic loss detected by in-vivo retinal imaging and CSF GFAP, NfL, NfH, may enable clinical trialing of TRPV4-targeted therapeutics (114, 115).

### 6.2 TRPV4 in Sensory Neurons and Pain

TRPV4 is robustly expressed in a subpopulation of small- to medium-diameter sensory neurons of the dorsal root ganglia (DRG) and trigeminal ganglia, where it functions as a polymodal detector of noxious osmotic, mechanical, thermal, and chemical stimuli (3). Immunohistochemical and single-cell RNA sequencing studies have co-localized TRPV4 with nociceptive markers including calcitonin gene-related peptide (CGRP), substance P, and the NGF receptor TrkA, but not with isolectin B4-binding non-peptidergic neurons, establishing its primary expression in peptidergic nociceptors (22).

The unambiguous demonstration of TRPV4 function in DRG neurons in vivo was achieved using Nav1.8-Cre-mediated conditional deletion of the *Trpv4* locus, which abrogated nocifensive responses to the endogenous TRPV4 activator lysophosphatidylcholine (LPC) without affecting TRPV4-dependent itch from keratinocytes (12). TRPV4 contributes to mechanical hyperalgesia in inflammatory pain states: intraplantar injection of inflammatory mediators such as carrageenan, complete Freund’s adjuvant, or the PAR2 agonist SLIGRL evokes mechanical hypersensitivity that is markedly attenuated in TRPV4 global and sensory-neuron-specific knockout mice, as well as by intrathecal or systemic TRPV4 antagonists (8, 16, 27, 38).

In neuropathic pain models, TRPV4 plays a well-substantiated role. Following peripheral nerve injury, TRPV4 expression increases in injured DRG neurons and adjacent satellite glial cells (165). Paclitaxel-induced painful peripheral neuropathy, a prevalent and dose-limiting chemotherapy complication, involves TRPV4 upregulation in DRG neurons, and TRPV4 antagonism prevents and reverses established mechanical allodynia in rodent models (166), Fig. 2. In a companion mechanism, paclitaxel sensitizes TRPV4 channels through direct effects on the channel or its lipid microenvironment, enhancing calcium responses to mechanical and chemical stimuli (167). TRPV4 also contributes to pain associated with temporomandibular joint disorders through its expression in trigeminal ganglion neurons innervating the joint capsule, where inflammatory mediators and mechanical strain converge on the channel (57).

In visceral pain, TRPV4 expression in colonic sensory neurons is upregulated in models of irritable bowel syndrome and inflammatory bowel disease, where it detects luminal distension and mediates visceral hypersensitivity (16, 27). TRPV4 antagonists reduce visceromotor responses to colorectal distension in preclinical models, positioning TRPV4 as a target for chronic visceral pain (16).

### 6.3 TRPV4 and Itch

**Figure 5:**
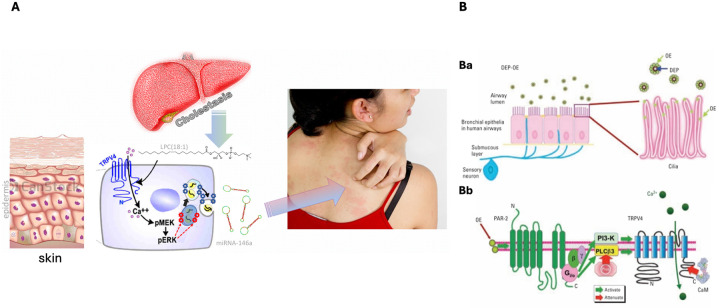
TRPV4 sentinel function in surface epithelia - cholestatic itch (internal danger signal) - Diesel exhaust particle exposure of human bronchial epithelia (external danger signal). A) Panel depicts schematically epidermal keratinocyte TRPV4 activation by excessive lysolipid, LPC, produced by cholestatic liver, which evokes keratinocyte exocytosis of miR-146a, a direct pruritogen that activates TRPV1+ pruriceptor sensory neurons to evoke chronic itch. Of note, this is a pervasive, serious clinical itch syndrome, yet without skin inflammation, demonstrating that metabolic disturbance can cause severe pruritus, without integumental inflamamtion, yet with key reliance of epidermal keratinocyte signaling. Based on (12). B) Panels depict airborne Diesel engine exhaust nanoparticles activating TRPV4, expressed at the brush border of human bronchial epithelia, to activate pro-inflammatory and pro-proteolytic effector mechanisms. Subpanel Ba shows schematic overview. Subpanel Bb shows signal transduction mechanisms, with co-involvement of PAR-2 signaling upstream of TRPV4 which then leads to calcium influx. Of note, PAR-2 functions here independent of proteolysis, and the effector principle of the Diesel exhaust particle is its organic chemical coating, not the particulate matter which function as delivery vehicle of an air pollution noxious agent. From (124).

**Figure 6:**
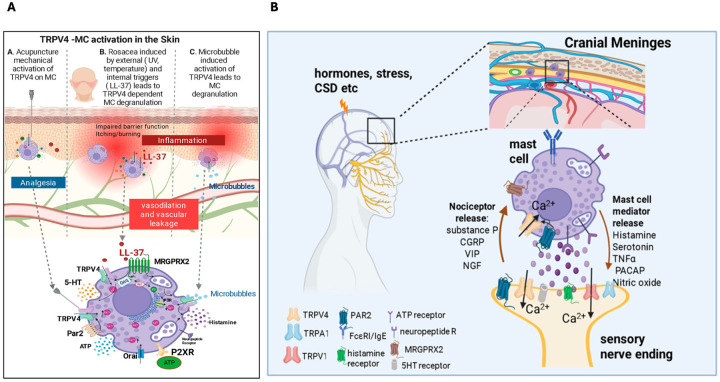
TRPV4 function in mast cells - cutaneous allergy, headache/migraine TRPV4 expressed and functional in mast cells that inhabit a stressed (inflamed) microenvironment leads to priming of these mast cells for vesicle release via TRPV4-mediated calcium influx. From (125). A) Panel depicts pro-inflammatory, pro-pruritic environment in skin e.g in eczema, but also histaminergic itch of parasite infestations. TRPV4's role in mast cells very likely contributes to amplification of pruritus via mast cell TRPV4. B) Panel depicts mast cells in the dural compartment of the skull, participating in migraine and headache pathogenesis by sensitizing trigeminal afferents that innervate the meninges where they further enhance the headache by neurogenic inflammation mediated secretion of pro-algesic peptides, CGRP and PACAP.

TRPV4 plays a central role in acute and chronic pruritus through mechanistically distinct contributions from epidermal keratinocytes, skin-resident macrophages, mast cells, neuroimmune circuits, and sensory neurons (149), Figs. 2, 5 and 6.

In keratinocytes, TRPV4 functions as a pruriceptor for histaminergic stimuli. Cell-type-specific conditional knockout demonstrated that keratinocyte TRPV4 is required for scratching evoked by histaminergic pruritogens, but not chloroquine, with calcium driving ERK phosphorylation as effector; topical TRPV4 inhibition recapitulated the genetic phenotype (150), Fig. 5. A second keratinocyte-centered contribution established lysophosphatidylcholine (LPC) as a cholestatic pruritogen acting via a defined C-terminal TRPV4 binding motif. Keratinocyte-*Trpv4* cKO mice showed markedly reduced LPC-evoked scratching, while sensory neuron-Trpv4 cKO mice were unaffected for the pruritogenic response but showed attenuated mechanical pain hypersensitivity following intrathecal LPC - establishing the first cell-type-specific separation of TRPV4-mediated itch versus nociception (12). The paracrine signal bridging keratinocytes to TRPV1^+^ pruriceptor neurons was identified as miR-146a, whose systemic levels correlated with itch intensity in primary biliary cholangitis patients, providing human clinical validation (12).

In mouse skin, keratinocyte and macrophage TRPV4 contribute differentially to chronic itch: keratinocyte TRPV4 can govern dry-skin itch, while macrophage TRPV4 is involved in allergic contact dermatitis itch, with platelet-derived serotonin contributing to both (151). An additional neuroimmune axis involves TRPV4-dependent TSLP production in keratinocytes (152).

In sensory neurons, TRPV4 mediates serotonin-evoked scratching (153) possibly forming functional heteromeric complexes with TRPV1 (154). Most recently, an IL-17R/ERK/TRPV4 axis in DRG/TG neurons was identified as a key mediator of psoriatic itch, providing mechanistic grounding for the anti-pruritic efficacy of IL-17-blocking biologics (155). Also recently, ATF4, not acting as transcription factor, was found to attenuate pro-pruritic impact of sensory neuronal TRPV4 in mouse and primate, and found to colocalize with TRPV4 in human DRGs (156).

Across all these contexts, topical TRPV4 antagonists have demonstrated preclinical anti-pruritic efficacy, and the convergence of cell-type-resolved mechanisms with human biomarker validation positions TRPV4 as an appealing therapeutic target for chronic pruritus.

## 7. Therapeutic Approaches: Toward Lineage-specific Targeting

The therapeutic targeting of TRPV4 requires careful consideration of the channel’s ubiquitous expression and the resulting significant risk of on-target adverse events that could derail therapeutics development programs. Small molecule selective inhibitors (e.g., GSK2193874, HC-067047, RN1734) have been developed, also potent dual TRPV4/TRPA1 inhibitors (16 − 8/16–19 compounds (128)), and molecular approaches including dominant-negative constructs and siRNA-mediated gene silencing. The orally active TRPV4 channel blocker GSK2193874 prevents and resolves pulmonary edema induced by heart failure in preclinical models (78). Actio Biosciences (San Diego CA) is developing compound ABS-0871 for treatment of hereditary TRPV4 channelopathies (168).

For disease contexts in which TRPV4 gain-of-function drives pathology—MS, TRPV4(mut) neurochannelopathies—endothelial-selective targeting of TRPV4 expression and function represents a future therapeutic strategy (121). This could be achieved by targeting CNS endothelial cells via ligand-based approaches using the transferrin receptor (TfR) or other endothelial surface markers such as CD36, VCAM1, and P-selectin, then delivering a TRPV4-targeted therapeutic (116). Recent advances in lipid nanoparticle (LNP) technology—deployed clinically for patisiran (siRNA targeting transthyretin, FDA-approved for TTR-amyloidosis neuropathy) and COVID-19 mRNA vaccines—offer a real-world-ratified platform for endothelial-specific TRPV4 silencing (117, 118). Combinatorial design of siloxane-incorporated LNPs and selective organ targeting (SORT) approaches enhance endothelial affinity and enable tissue-specific delivery (119, 120).

## 8. Personal Reflections on the Discovery of TRPV4

On a personal note, TRPV4 was on my benchtop in the Friedman Lab at The Rockefeller University in the late 1990s. That experience led to the title of this Chap. (1, 2). I sensed excitement in the lab’s air during those days, losing sleep about novelty. The entire world did not yet know about it, and there was a looming sentiment that others might preempt. When the paper appeared in *Cell* in October 2000, it reported VR-OAC/TRPV4 as a candidate vertebrate osmoreceptor with a potential to function in mechanical signal transduction as well.

Fast forward to today, it is rewarding to reflect on how far our understanding of TRPV4 has evolved, and to be able to share thoughts on TRPV4’s role in multiple human diseases characterized by unmet medical need. The channel we first characterized as an osmotic sensor now touches nearly every organ system and disease category covered in contemporary biomedical research. Many areas have benefited from TRPV4-based discoveries, from demonstrating the evolutionarily conserved role of TRPV4 in *C. elegans* osmo-mechanotransduction (4, 24), to trigeminal pain (65), skin barrier biology (7, 12), skeletal mechanosensing (60–62), central osmoregulation (25), and most recently, endothelial TRPV4 function in obesity hypertension (54), multiple sclerosis (69), and hereditary neuro-channelopathies (100). In bibliometric terms, the field now nears 3,000 publications across more than 50 organ-specific and disease-specific domains, with contributions from hundreds of laboratories worldwide, indicating that a chapter as presented here represents a heartwrenching challenge for its author - because it has to short-cut (too) many extra-ordinary TRPV4-related discoveries that were made along the path toward current understanding.

## 9. Methods - Systematic Review Methodology and Criteria

### Literature Search Strategy and Eligibility.

**Figure 7:**
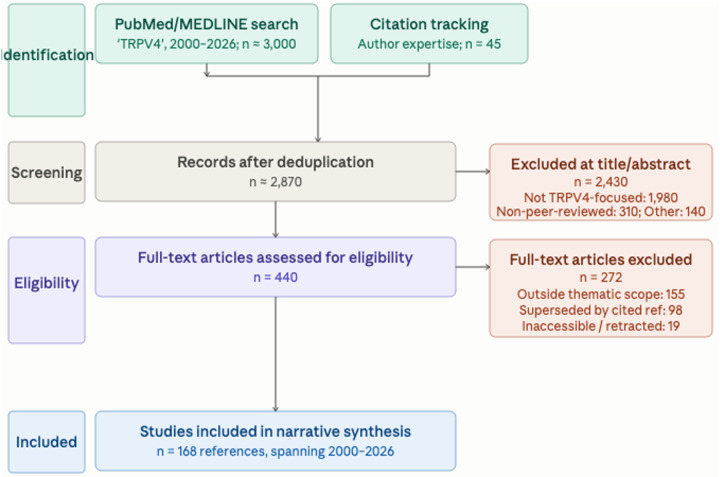
PRISMA exclusion flowchart underpinning Systematic Review Primary source: PubMed/MEDLINE | Search term: “TRPV4”, “OTRPC4”, “VRL1”, “TRP12” | Date range: October 2000 – May 2026

This review was conducted as a systematic survey of the primary and review literature on transient receptor potential vanilloid member 4 (TRPV4) published since the channel's discovery. A systematic search of PubMed/MEDLINE was performed using the search terms “TRPV4”, “OTRPC4”, “VRL2” and “TRP12”, with no restriction on field or subheading, covering the period from October 2000 (the date of the first TRPV4 cloning reports) through May 2026. This search yielded approximately 3,000 records. An additional 45 records were identified through forward and backward citation tracking of landmark papers and through the author's expert knowledge of the field. Following removal of duplicates, 2,870 records were screened at the title and abstract level. Records were excluded if TRPV4 was mentioned only incidentally without expressional or functional data (n = 1,980), if the source was non-peer-reviewed or a conference abstract (n = 310), or if the report was non-English or inaccessible in full text (n = 140). A total of 440 full-text articles were assessed for eligibility. Of these, 272 were excluded because they fell outside the 17 thematic domains covered in this review (n = 155), were superseded by a more definitive, directly cited publication (n = 98), or were retracted or otherwise inaccessible (n = 19). The final synthesis includes 168 references; a PRISMA-compliant exclusion flowchart is shown in Fig. 7, inclusion- and exclusion criteria in Table 1.

### Evidence Synthesis.

Because TRPV4 biology spans more than 50 organ-specific and disease-specific domains, this review employs a structured narrative synthesis rather than a single pooled meta-analysis. The 168 included studies are organized into 17 thematic domains (Table 2) and appraised with respect to study design (in vitro, preclinical in vivo, human translational, or human genetic), model organism, primary TRPV4 role identified, and translational stage. Quantitative effect estimates, where reported in the primary literature (for example, changes in vascular tone, survival rescue from chemotoxicity, intraocular pressure reduction), are reproduced in the relevant sections alongside the original study's statistical descriptors. No statistical pooling across studies was performed, as the heterogeneity of experimental endpoints, species, and organ systems precludes a meaningful aggregate effect size. This approach aligns with PRISMA-ScR (Preferred Reporting Items for Systematic Reviews and Meta-Analyses -- Scoping Reviews) guidance for comprehensive evidence mapping of a multisystem biological target.

## Figures and Tables

**Table 1:** Inclusion and Exclusion Criteria Criteria applied during systematic search and screening of the TRPV4 literature (PubMed/MEDLINE, 10/2000–5/2026).

Inclusion criteria	Exclusion criteria
**INCLUDE**: Peer-reviewed original research articles, systematic or narrative reviews, genetics reports, and case series.	**EXCLUDE**: Conference abstracts, preprints, letters, and non-peer-reviewed commentary.
**INCLUDE**: Studies in which TRPV4 is the primary focus: expression documented, function measured, or pharmacology reported.	**EXCLUDE**: Studies mentioning TRPV4 incidentally, without quantitative expressional or functional data.
**INCLUDE**: Publication date October 2000 to May 2026, spanning the full post-discovery era of TRPV4 research.	**EXCLUDE**: Reports antedating the cloning of TRPV4/VR-OAC (pre-October 2000), except foundational TRP channel biology.
**INCLUDE**: Any study design: in vitro, ex vivo, preclinical in vivo (any species), human genetics, or clinical translational.	**EXCLUDE**: Purely computational or in silico studies lacking independent experimental validation of TRPV4 activity.
**INCLUDE**: Any model organism: Homo sapiens, Mus musculus, Rattus norvegicus, Danio rerio, Caenorhabditis elegans, avian, porcine.	**EXCLUDE**: Studies in which results were fully superseded by a more definitive, directly cited later publication.
**INCLUDE**: English-language publications with accessible full text.	**EXCLUDE**: Non-English language; inaccessible full text; retracted articles.
**INCLUDE**: Topics spanning any organ system or disease category in which TRPV4 expression or function is characterized.	**EXCLUDE**: Topics outside the 17 thematic domains covered in the present review.
**INCLUDE**: Therapeutic studies (small molecules, genetic tools, nanoparticle delivery) where TRPV4 is the primary target.	**EXCLUDE**: Drug or gene-therapy studies where TRPV4 is not the primary pharmacological or molecular target.

**Table 2:** Quantitative Synthesis by Thematic Domain Domain-level evidence map of 168 included references across 17 thematic domains.

Thematic domain	Refs (n)	Predominant study types	Primary TRPV4 role identified	Translational stage
**Basic biology**				
**Channel discovery, structure & function**	**21**	In vitro; biophysics; cryo-EM; heterologous expression	Polymodal Ca2+-permeable nonselective cation channel; homotetramer; ankyrin repeats; AKAP150 scaffold	Foundational
**Invertebrate orthology (C. elegans / OSM-9)**	**5**	In vivo (worm); behavioural genetics; mutagenesis	Osmotic/mechanical sensing conserved across evolution; Ca2 + vs Na+ permeation governs habituation vs acute avoidance	Foundational
**Neurological and sensory**				
**Neurobiology & neuroprotection**	**10**	In vitro; mouse KO/KI; DRG cultures	Osmosensory; astrocytic volume/BBB regulation; neuroprotection vs CIPN and soman neurotoxicity	Preclinical
**Pain (inflammatory, trigeminal, neuropathic, visceral)**	**15**	Mouse KO/KI; behavioural assays; ex vivo nerve fibres	Pro-nociceptive; PAR-2 downstream sensitization; mechanical hyperalgesia; visceromotor responses	Preclinical -- therapeutic target
**Itch -- acute and chronic pruritus**	**12**	Mouse conditional KO; human cholangitis biomarkers	Pruriceptor in keratinocytes (LPC/histamine); itch-nociception dissociation; psoriatic itch via IL-17R/TRPV4	Preclinical -- biomarker-validated (human)
**Inflammatory and immune**				
**Inflammation & immunity**	**9**	Macrophage/neutrophil assays; rodent models	Macrophage phagocytosis and stiffness-polarization; mechanosensing-immunity link; BBB immune trafficking	Preclinical
**Tuberculosis & infectious disease**	**4**	Mtb-infected mouse; macrophage cultures	Biphasic: acute host-protective (lysosomal maturation) then chronic immunopathological; antiviral via DDX3X (dengue, HCV, Zika)	Preclinical
**Cardiovascular and pulmonary**				
**Vascular biology, heart & MS endothelia**	**26**	Endothelial cell assays; mouse; human arterioles; MS brain sections	Ca2 + sparklets driving vasorelaxation; obesity hypertension via NOX1- peroxynitrite; MS BBB leakage; BSCB failure in channelopathy	Human-validated (obesity, MS, channelopathy)
**Lung & airway biology**	**14**	Epithelial/endothelial cells; rodent lung-injury models	Alveolar barrier disruption; pulmonary edema; hypoxic vasoconstriction; CF defect; diesel exhaust particle injury	Preclinical -- 1 compound (GSK2193874) in development
**Epithelial and organ-specific**				
**Skin biology & rosacea**	**10**	Keratinocyte assays; mouse KO; human skin biopsy	Sunburn pain; barrier function; rosacea via LL37/mast cells; wound healing; scleroderma myofibroblast differentiation	Preclinical -- topical targeting feasible
**Gastrointestinal tract**	**7**	Rodent colitis/pancreatitis models; organoids	Visceral hypersensitivity (IBS/IBD); pancreatitis pain; NAFLD resistance via eNOS; GLP-1 release via NCX1	Preclinical
**Renal & urinary biology**	**4**	Tubular cell assays; mouse KO	Distal nephron mechano-osmosensor; TRPP2 heteromer; cystogenesis in ARPKD; overactive bladder	Preclinical
**Uterine function & preterm birth**	**3**	Mouse; human myometrial strips	Osmo-mechanosensor driving contractility; TRPV4 blockade quells preterm labor in human-derived tissue	Human-tissue validated; tocolytic target
**Fluid homeostasis (CSF, aqueous humor, endolymph)**	**6**	Epithelial cell assays; rodent models	Choroid plexus CSF secretion; trabecular meshwork -- IOP/glaucoma; stria vascularis -- endolymph	Preclinical -- glaucoma target
**Metabolic and endocrine**				
**Metabolism, obesity & diabetes**	**9**	Adipose KO mouse; pancreatic beta-cell assays	Adipose thermogenesis/inflammation; GLP-1 secretion via NCX1; insulin secretion; vasopressin/renin regulation	Preclinical
**Skeletal and connective tissue**				
**Skeletal biology -- bone, cartilage & joints**	**16**	Chondrocyte/osteoclast assays; mouse cartilage-specific KO	Sixth sense of musculoskeletal system; chondrocyte loading response; osteoarthritis; GCTB; osteonecrosis	Preclinical -- OA therapeutic target
**TRPV4 channelopathies -- skeletal & neural**	**17**	Human genetics (GoF mutations); humanized KI mouse	Brachyolmia; metatropic dysplasia; CMT2C; dSMA; endothelial BSCB failure drives motoneuron degeneration	Human genetic disease -- ABS-0871 in development
**Mechanogenetics & tissue engineering**	**6**	Stem cell gene-circuit constructs; ex vivo cartilage	Mechanically triggered gene circuit for on-demand therapeutic delivery; TRPV4 as synthetic input transducer	Proof-of-concept (preclinical)
**Cancer and therapeutics**				
**Cell growth & cancer biology**	**4**	Cancer cell lines; xenograft models	Candidate co-oncogene; migration/invasion; tumor angiogenesis; mechanosensing in stiff tumor ECM	Preclinical (inhibit vs activate dilemma)
**Therapeutic strategies & LNP delivery**	**10**	Pharmacology; rodent; human endothelial cells	GSK2193874 (pulmonary edema); dual TRPV4/TRPA1 inhibitors (16–8/16–19); ABS-0871 (channelopathy); SORT-LNP endothelial silencing	2 compounds in clinical/regulatory development
**TOTAL**	**168**	**17 thematic domains | 2000–2026 | species: human, mouse, rat, worm, guinea pig, zebrafish**		

a GoF = gain-of-function; KO = knockout; KI = knock-in; cKO = conditional knockout; IBS = irritable bowel syndrome; IBD = inflammatory bowel disease; NAFLD = non-alcoholic fatty liver disease; CIPN = chemotherapy-induced peripheral neuropathy; ARPKD = autosomal recessive polycystic kidney disease; GCTB = giant cell tumor of bone; BBB = blood-brain barrier; BSCB = blood-spinal cord barrier; MS = multiple sclerosis; CMT2C = Charcot-Marie-Tooth type 2C; dSMA = distal spinal muscular atrophy; LNP = lipid nanoparticle; SORT = selective organ targeting.

b n values reflect the number of references in this review primarily assigned to each domain. Some references contribute to more than one domain; the total is therefore non-additive.

c Translational stage: Foundational = mechanism characterization; Preclinical = validated in animal disease model; Human-tissue validated = tested in human-derived cells or explants; Human-validated = data from human subjects or confirmed in clinical disease.
